# Behcet's Disease with Upper GI Bleeding

**DOI:** 10.1155/2020/2983209

**Published:** 2020-03-17

**Authors:** Melaku Getachew, Nebiyou Seyoum, Finot Debebe

**Affiliations:** ^1^Department of Emergency Medicine, Addis Ababa University, Addis Ababa, Ethiopia; ^2^Department of Surgery, Addis Ababa University, Addis Ababa, Ethiopia

## Abstract

*Introduction*. Behcet's disease is a multisystem disease. In sub-Saharan Africa, the prevalence of this disease is not known, with only one case report from Ethiopia. *Case Presentation*. We describe a case of a 29-year-old Ethiopian male who presented to the emergency room of Tikur Anbessa specialized hospital with 4 days history of back pain, recurrent history of oral and genital ulcers, right eye blindness, chronic cerebral vein thrombosis, gastrointestinal bleeding, aortic aneurysm with dissection, and positive pathergy test. He is retrospectively diagnosed with Behcet's disease according to both the International Criteria for Behcet's Disease (ICBD) and the International Study Group (ISG) consensus. *Conclusion*. Even if Behcet's disease is rare in sub-Saharan Africa, it is important to know the clinical presentation for timely diagnosis and urgent management.

## 1. Introduction

Behcet's disease (BD), also called as a Bechet's syndrome, is manifested by the recurrent oral ulcers and other systemic manifestations which involves the skin, eye, and cardiovascular, pulmonary, and neurologic systems [1–4]. The pathogenesis of Behcet's disease is not known. It is a variable-sized vessel vasculitis with polygenic genetic predisposition usually triggered by infectious agent [1]. The prevalence of Behcet's disease in Ethiopia is not known, and only one case was reported from Ethiopia in 1997 [2]. Usually, BD is diagnosed in young adults (20–35 years), with no difference between sexes [5].

## 2. Case Report

We report a 29-year-old Ethiopian male patient who presented to the emergency room of Tikur Anbessa specialized hospital with 4 days history of persistent interscapular back pain, which is of stabbing nature and radiating to the anterior chest bilaterally. He also had persistent high-grade fever, vomiting for ingested matter, easy fatigability, and difficulty in swallowing.

He also complained of painful recurrent oral ulcers in the past 5 years (three recurrences of mouth ulcer in the past 1 year), scrotal ulcer of 2 years, and right side eye blindness of 1 year duration. It started gradually from the buccal cavity of the mouth and inner side of the lower lip. It completely healed and recurred three times in the past one year. The scrotal and penile shaft ulcers were pus discharging.

The past history was significant for chronic superior sagittal and sigmoid sinus thrombosis 9 years back. For this, he was on warfarin and discontinued the medication 4 months prior to current presentation for unspecified reasons. He had also repeated history of treatment for sexually transmitted infections.

Otherwise, he did not have previous history of abdominal pain, yellowish discoloration of eyes, body swelling, and reddish discoloration of urine, dysuria, or change in urine amount. He has no previous history of sexual transmitted diseases. He was admitted to hospital several times for the above compliant. He had been treated with muconazol gel and several courses of antibiotics, but there was no improvement.

On arrival to the emergency room, physical examination revealed an acutely sick looking young man in pain. His vital signs were as follows: blood pressure was 100/70 mm Hg; heart rate was 104 beats per minute regular and full in volume; respiratory rate was 36 breaths per minute and oxygen saturation was 95% at atmospheric air. Pertinent findings were on HEENT and musculoskeletal and nervous systems. He had scattered whitish superficial ulcers on the ventral side of the tongue and buccal surface of the lower lip. He had healed old scar on the penile shaft and active pus discharging ulcer at the base of the penile shaft and under the surface of the scrotum with scattered pustules. The nervous system evaluation revealed bilaterally pale and mildly atrophic tongue with right side corneal opacity. Other physical examinations were unremarkable.

### 2.1. Blood Work

Complete blood count showed leukocytosis of 15,540 cells (72.3% neutrophils and 19.2% lymphocytes), and the other cell lines were normal. Serology test of VDRL for syphilis and HIV rapid test were negative. Gram staining and AFB from scrotal ulcer swab were negative. Random blood glucose level was 124 mg/dl, and ESR was 80 mm/HR. Blood chemistry, cardiac biomarkers, serum electrolytes, and ECG were normal. Pathergy test was positive.

### 2.2. Imaging

A chest CT scan with contrast showed right lateral proximal descending aorta secular outpouching with narrow neck measuring 4.8 cm with surrounding intramural hematoma with mass effect on the esophagus. There was no evidence of aortic dissection.

### 2.3. Management

The patient was directly taken from the triage to the resuscitation area. He was put on the monitor. Intravenous line was secured, and he received morphine syrup (4 mg IV every 4 hours and subsequently escalated to continuous infusion 4 mg/HR.), metoprolol (5 mg IV stat with maintenance 25 mg PO BID, which was subsequently escalated to 50 mg PO QID), vancomycin (1 g IV BID), cefepime (2 g IV TID), and cimetidine (200 mg IV BID).

After 2 days of emergency department stay, he developed worsening of dysphagia and bloody vomiting with an estimated loss of 500 ml of blood. He was treated with omeprazole (80 mg IV stat) and resuscitated with 1 unit of whole blood and 3 liter of normal saline. Subsequently, he was taken to the operating room with the impression of aortoesophageal fistula. He received ketamine (25 mg IV) for pain control. Left posteriolateral thoracotomy was performed. Intraoperatively, there was a 5 × 4 cm proximal descending aortic aneurysm just distal to the origin of the left subclavian artery with adhesion and fistulization to mid-esophagus posteriorly. Aortic aneurysmorhaphy (primary repair) was performed, and there was a mid-esophageal 3 cm necrosis and fistula formation which was repaired over a nasogastric tube. A chest tube was inserted on the left side. The patient was transferred to intensive care unit, and his immediate postoperative course was smooth. He was transferred to regular ward on the 3^rd^ day.

On his 8^th^ postoperative day, the patient developed severe retrosternal chest pain and chest X- ray was taken, and it reveled significant mediastinal widening. Unfortunately, the patient passed away on the 9^th^ postoperative day after he developed massive upper gastrointestinal bleeding in the surgical ward.

### 2.4. Diagnosis

The diagnosis of Bechet's disease in our patient was made retrospectively according to the International Criteria for Bechet's Disease (ICBD) [6] based on genital ulcer (two points), ocular lesions (two points), oral ulcer (one point), skin manifestations (one point), vascular lesions (one point), and pathergy test (one point, done postoperatively). For diagnosis of Bechet's disease, the ICBD recommends at least three points, and the patient fulfilled all criteria (eight points), as shown in Table 1.

## 3. Discussion

Behcet's disease is a variable-sized vessel vasculitis which involves different systems. It presents with recurrent oral and genital ulcerations and ocular involvement. The prevalence of BD in Ethiopia is not known, and only one case was reported from Ethiopia and only a few cases were reported from sub-Saharan countries [1, 2, 7]. We believe that Behcet's disease remain misdiagnosed or remain undiagnosed due to unawareness regarding disease among health professionals in these countries. There was a delay in diagnosis of Behcet's disease, as shown in this case (9 years) and other similar case reports (5.5 and 2 years, respectively, in Comoros and Nigeria) [1, 7]. The patient had a typical presentation for BD which fulfills the criteria of the ICBD and International Study Group for Behcet's disease criteria [6], but he was not diagnosed early and was repeatedly treated for sexual transmitted infections.

Behcet's disease can reach serious proportions without early diagnosis and treatment [2, 7, 8]. Almost all patients of BD present with lesions of painful nonscarring oral mucocutaneous aphthous ulcerations [3, 5]. Eye involvement with scarring and bilateral panuveitis is the most serious complication which occasionally progresses rapidly to blindness if untreated early [3]. Vascular manifestations with thrombosis, DVT, are seen in 30% of patients, and rarely with vascular aneurysm and dissection (5–10%). The central nervous system, especially brain stem, involvement is associated with serious prognosis [3].

This patient was not treated appropriately for the eye complaints and later on presenting with blindness. He also developed other complications like cerebral venous thrombosis, aortic aneurysm, upper gastrointestinal bleeding, and ended up dying.

Surgical management for BD in the fulminant or in the acute phase will increase patient mortality and morbidity. Bleeding from suture lines and pseudoaneurysm formation in the early and late phase is high retrospectively. It is better to treat patients with glucocorticoids in the pre- and postoperative periods [9, 10].

## 4. Conclusion

We believe that the report of this clinical case can be useful to develop better knowledge or awareness to health professionals working in sub-Saharan Africa, especially in Ethiopia, on BD so as to early diagnose and urgent treatment with glucocorticoids and immunosuppressive drugs. Additional studies are recommended to know the prevalence of BD in Ethiopia and sub-Saharan countries. We also suggest that pre- and postoperative glucocorticoid and immunosuppressive treatment will determine the survival and prevention of complication of BD.

## Figures and Tables

**Table 1:** International Criteria for Bechet's Disease (ICBD) point score system: scoring 3 or more indicates Bechet's disease [4].

Sign/symptom	Points
Genital ulcer	Two
Ocular lesions	Two
Oral ulcer	One
Skin manifestations	One
Vascular lesions	One
Positive pathergy test	One
